# A Broad-Spectrum Antiviral Peptide Blocks Infection of Viruses by Binding to Phosphatidylserine in the Viral Envelope

**DOI:** 10.3390/cells9091989

**Published:** 2020-08-29

**Authors:** Rutger D. Luteijn, Patrique Praest, Frank Thiele, Saravanan Manikam Sadasivam, Katrin Singethan, Jan W. Drijfhout, Christian Bach, Steffen Matthijn de Boer, Robert J. Lebbink, Sha Tao, Markus Helfer, Nina C. Bach, Ulrike Protzer, Ana I. Costa, J. Antoinette Killian, Ingo Drexler, Emmanuel J. H. J. Wiertz

**Affiliations:** 1 Department of Medical Microbiology, University Medical Center Utrecht, 3508 GA Utrecht, The Netherlands; rdluteijn@gmail.com (R.D.L.); p.praest-2@umcutrecht.nl (P.P.); R.J.Lebbink-2@umcutrecht.nl (R.J.L.); A.I.CorreiadeAlmeidaCosta@umcutrecht.nl (A.I.C.); 2 Institute of Virology, Technical University and Helmholtzzentrum Munich, 81675 Munich, Germany; frank.thiele@helmholtz-muenchen.de (F.T.); katrin.singethan@tum.de (K.S.); ch-bach@gmx.de (C.B.); helfer.markus@gmail.com (M.H.); protzer@tum.de (U.P.); 3 Membrane Biochemistry and Biophysics, Bijvoet Centre for Biomolecular Research, Utrecht University, 3584 CH Utrecht, The Netherlands; mssaravananbt@gmail.com (S.M.S.); J.A.Killian@uu.nl (J.A.K.); 4 Department of Immunohematology and Blood Transfusion, Leiden University Medical Center, 2333 ZA Leiden, The Netherlands; j.w.drijfhout@lumc.nl; 5 Virology Division, Department of Infectious Diseases & Immunology, Faculty of Veterinary Medicine, Utrecht University, 3584 CL Utrecht, The Netherlands; matthijn.de.boer@intravacc.nl; 6 Institute for Virology, University Hospital Düsseldorf, Heinrich-Heine-University, 40225 Düsseldorf, Germany; sha.tao@med.uni-duesseldorf.de (S.T.); Ingo.Drexler@med.uni-duesseldorf.de (I.D.); 7 Institute of Chemistry, Chair of Organic Chemistry II, TU Munich, 85748 Garching, Germany; nina.bach@tum.de

**Keywords:** antiviral peptide, enveloped viruses, membrane phosphatidylserine, envelope disruption

## Abstract

The ongoing threat of viral infections and the emergence of antiviral drug resistance warrants a ceaseless search for new antiviral compounds. Broadly-inhibiting compounds that act on elements shared by many viruses are promising antiviral candidates. Here, we identify a peptide derived from the cowpox virus protein CPXV012 as a broad-spectrum antiviral peptide. We found that CPXV012 peptide hampers infection by a multitude of clinically and economically important enveloped viruses, including poxviruses, herpes simplex virus-1, hepatitis B virus, HIV-1, and Rift Valley fever virus. Infections with non-enveloped viruses such as Coxsackie B3 virus and adenovirus are not affected. The results furthermore suggest that viral particles are neutralized by direct interactions with CPXV012 peptide and that this cationic peptide may specifically bind to and disrupt membranes composed of the anionic phospholipid phosphatidylserine, an important component of many viral membranes. The combined results strongly suggest that CPXV012 peptide inhibits virus infections by direct interactions with phosphatidylserine in the viral envelope. These results reiterate the potential of cationic peptides as broadly-acting virus inhibitors.

## 1. Introduction

The continuous threat of conventional viruses and emergence of new virus species requires an ongoing search for new antiviral compounds. Broad-spectrum antiviral inhibitors have become promising therapeutic candidates [1]. These compounds act on common elements shared by many groups of viruses, including the synthesis of viral RNA and DNA, viral proteases, and glycosylation of viral proteins (reviewed by [2]). An important class of broad-spectrum antivirals targets the lipid envelope of viruses [3,4,5,6,7]. These include not only antibodies and chemical compounds, but also cationic peptides present as defense peptides in the human host and other organisms. In essence, the applicability of such peptides as antiviral compounds targeting the envelope of viruses will depend on their ability to discriminate viral from human membranes.

Enveloped viruses, in contrast to non-enveloped ones, have their nucleocapsid surrounded by a lipid membrane. Depending on the virus, this lipid bilayer is derived from the plasma membrane or intracellular organelles. Although the membrane is host cell-derived, it may differ functionally and structurally from the membrane of origin in several aspects, including the lipid composition. Compared to host cell membranes, the envelope of many viruses is enriched for the phospholipid phosphatidylserine (PS). PS is the most abundant anionic lipid in eukaryotic cells. In resting cells, PS is mainly contained within the cell, with only very limited exposure to the extracellular environment. PS is enriched in some intracellular organelles, but the majority of PS is asymmetrically distributed on the inner leaflet of the plasma membrane [8]. This organization results from constantly flipping PS from the outer membrane leaflet to the inner membrane leaflet by ATP-dependent flippases. As a result, the outer exposed membrane leaflet of the cellular plasma membrane is devoid of PS. In apoptotic cells, this organization is lost due to the inactivation of flippases and the activation of scramblases. The resulting PS exposure on apoptotic cells and cell debris induces uptake by surrounding cells through PS receptors. Many viruses take advantage of this uptake mechanism by exposing PS on their envelope, thereby facilitating virus entry. The increase in PS on the virus membrane may be due to the lack of an active flippase. Alternatively, some viruses actively accumulate PS at sites of virus budding, or bud from subcellular microdomains enriched in PS [9].

Another important aspect of the host membrane is its property of self-renewal upon injury. This fast-acting mechanism involves several events, including the detection of the damaged site, exocytosis of nearby cytosolic vesicles, cytoskeletal remodeling, endocytosis of the damaged membrane, and reconstitution of membrane homeostasis [10,11]. The lipid membrane loses its self-renewal capacity once surrounding the viral nucleocapsid, thereby becoming particularly prone to membrane damage [5]. The structural and functional differences between virus and host cell membranes make viral membranes ideal targets for antiviral therapy.

Here, we identify a peptide with broad-spectrum antiviral activity, whose sequence is derived from the cowpox virus protein CPXV012. Within virus-infected cells, this protein helps to evade the immune system by inhibiting the transporter associated with antigen processing (TAP), thereby interfering with MHC I-dependent antigen presentation [12,13,14,15,16]. We pinpointed the segment of the CPXV012 protein responsible for blocking TAP: a peptide comprising the C-terminal domain of CPXV012. It should be noted that we have no indication that this peptide is produced upon CPXV infection. Interestingly, when doing functional assays with this peptide, upon infection we noticed that the percentage of infected cells decreased significantly. This finding was the basis for the current study. Here, we show that this peptide inhibits infection of many enveloped viruses by interacting with virus particles. Variants of the CPXV012 peptide revealed that basic residues within the peptide are important for this inhibitory effect. Furthermore, CPXV012 strongly interacts with lipid monolayers and membranes enriched for the anionic phospholipid PS. These results suggest that this CPXV012 peptide disturbs the integrity of viral membranes enriched for PS, and thus may be explored as an antiviral agent against a broad range of enveloped viruses.

## 2. Materials and Methods

### 2.1. Cells and Viruses

MelJuSo (MJS) and BHK21 cells were cultivated with RPMI 1640 containing 10% FCS, 100 U/mL penicillin, 100 µg/mL streptomycin, and 2 mM L-glutamine (complete medium). HEK293T, HeLa, Huh7.5, and Vero cells were grown in DMEM supplemented with 10% FCS (5% for Vero cells), 100 U/mL penicillin, 100 µg/mL streptomycin, 1% nonessential amino acids, 2 mM L-glutamine, and 1% sodium pyruvate (complete DMEM). HepRG cells were differentiated in Williams E medium supplemented with 10% FCS (Fetalclone II from Hyclone, Thermo Scientific; Breda, The Netherlands), 100 U/mL penicillin, 100 µg/mL streptomycin, glutaMax, 0.023 IE/mL human insulin (Sanofi Aventis, Amsterdam, Netherlands), 4.7 µg/mL hydrocortisone (Pfizer, Capelle a/d IJssel, The Netherlands), 80 µg/mL Gentamicin, and 1.8% DMSO. HepG2.2.15 cells were cultivated in Williams E medium with 10% FCS, 100 U/mL penicillin, 100 µg/mL streptomycin, and 1% nonessential amino acids. LC5-RIC cells (EASY-HIT assay) were maintained as described previously [17].

Recombinant modified vaccinia virus Ankara (MVA) expressing eGFP under the natural early/late promoter p7.5 was used in this study (MVA-eGFP) [18]. MVA-eGFP was propagated and titrated in chicken embryonic fibroblasts (CEFs) according to standard methodology [19]. Vaccinia virus expressing eGFP (VACV-eGFP) was a generous gift from Dr. Jon Yewdell (NIH, Bethesda, USA). VACV-eGFP was propagated and titrated on CV-1 cells. Cowpox virus strain Brighton Red (CPXV-BR) expressing RFP/eGFP virus was originally obtained from Dr. Karsten Tischer (FU Berlin, Germany). HSV-1 expressing the VP16-eGFP was kindly provided by Dr. Peter O’Hare (Imperial College London, UK). Stocks were prepared and titrated on Vero cells.

HBV particles were concentrated from the supernatant of HepG2.2.15 cells as described previously [20]. Infectious HIV-1 stocks were prepared as described before [17]. Measles virus expressing eGFP (MV-eGFP) was generated as previously described [21]. Vesicular stomatitis virus expressing Luciferase (VSV-deltaG (Luc)) was a kind gift from Dr. Gert Zimmer (Institute of Virology and Immunology, Mittelhäusern, Switzerland). Recombinant adenovirus expressing GFP and ovalbumin (AdGOva) was kindly provided by Dr. Percy Knolle (Institutes of Molecular Medicine and Experimental Immunology, Bonn, Germany) and virus stocks were prepared as previously described [22]. Non-spreading Rift Valley fever virus (RVFV) replicon particles were produced and titrated as described previously [23]. Coxsackie B3 virus expressing Renilla Luciferase (RLuc-CVB3) was produced and titrated as described previously [24].

### 2.2. Peptides

The inhibitory peptide used in this study comprises amino acids 36 to 69 of the CPXV012 protein (QEGISRFKICPYHWYKQHMSLLFRRYYHKLDSII). The control peptide is derived from the pseudorabies virus TAP inhibitor UL49.5 and comprises amino acid residues 28 to 59 of this protein (sequence: STEGPLPLLREESRINFWNAAUAARGVPVDQP). U in amino acid sequence refers to alpha-amino-n-butyric acid, see below.

Synthetic peptides were prepared by normal Fmoc chemistry using preloaded Tentagel resins, using PyBop/N-methylmorpholine for in situ activation and 20% piperidine in N-methylpyrrolidinone for Fmoc removal [25]. Couplings were performed for 75 min. After final Fmoc removal, peptides were cleaved with trifluoroacetic acid/H2O 19:1 *v/v* containing additional scavengers when C or W residues were present in the peptide sequence. Peptides were isolated by ether/pentane precipitation. Peptides were checked for purity using reversed-phase HPLC–mass spectrometry and for integrity using MALDI-TOF mass spectrometry, showing the calculated molecular masses. All peptides were synthesized with an N-terminal biotin moiety and a C-terminal amide. Cysteines were replaced by the isosteric alpha-amino-n-butyric acid. Lyophilized peptides were dissolved in DMSO. Peptide concentrations were confirmed by the NanoDrop spectrophotometer (Thermo Scientific, Amsterdam, The Netherlands) using the absorbance of Trp at 280 nm. Peptides were aliquoted in Eppendorf LoBind microcentrifuge tubes (Sigma-Aldrich, Zwijndrecht, The Netherlands) and kept at −20 °C for short-term storage.

### 2.3. Cell Viability Assays

Cells were incubated with CPXV012 peptide, control peptide, or DMSO at the indicated concentrations for at 37 °C for 20 to 24 h. Thereafter, cell viability was measured using different assays. DAPI staining was performed to discriminate between live and dead cells using flow cytometry. Cell Titer-Blue Cell Viability Assay (Promega, Leiden, The Netherlands) was performed according to the manufacturer’s instructions. Neutral Red uptake (Applichem, Darmstadt, Germany) was performed as described previously [26]. WST-1 salt conversion assay (Roche, Woerden, The Netherlands) was performed according to the manufacturer’s instructions at 7 or 20 h post exposure to the peptides. No difference was observed between 7 and 20 h post exposure.

### 2.4. Virus Inhibition Assays

#### 2.4.1. MVA, VACV, and Cowpox

MJS, BHK21, HeLa, and HEK-293T cells (1 × 10^5^) were seeded in 24-well plates in a total volume of 400 µL and incubated overnight at 37 °C. Cells were infected with MVA-eGFP, VACV-eGFP, or CPXV-RFP/eGFP at 37 °C for 18 to 20 h in medium containing 0.1% DMSO and the indicated concentrations of either CPXV012 peptide or control peptide UL49.5 or 0.1% DMSO only as vehicle control. Flow cytometric analysis was performed to quantify the percentage of infected cells as indicated by eGFP or RFP expression.

Where mentioned, MVA-eGFP was pretreated with 50, 100, or 150 µg/mL CPXV012 peptide, control peptide, or DMSO vehicle at 37 °C for 1 h. Thereafter, the virus-CPXV012 peptide mixture was diluted 1:10 by volume and used to infect MJS cells (corresponding to an MOI of 10) resulting in a final concentration of 5, 10, or 15 µg/mL peptide in the culture medium. As a control, MJS cells were incubated with the same concentrations of CPXV012 peptide (5, 10, and 15 µg/mL) during infection with MVA-eGFP that was not pretreated with peptides. After 20 h, the cytometric analysis was performed to quantify the percentage of infected cells indicated by eGFP-expression.

**Figure 1:**
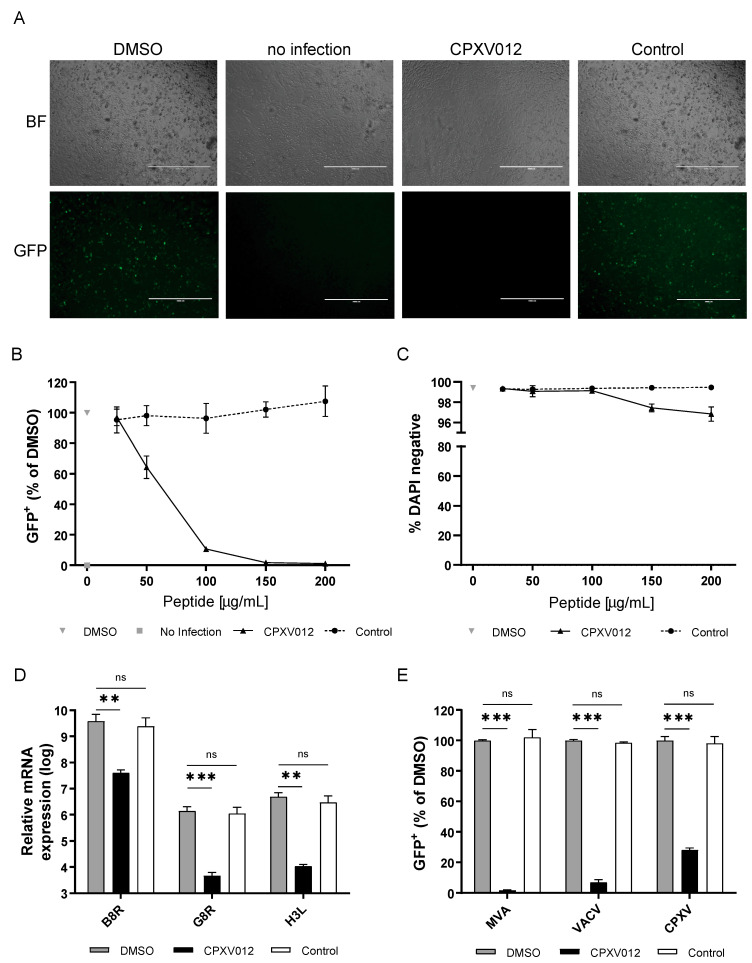
CPXV012 peptide inhibits poxvirus infection. (**A**) MJS cells were infected with MVA-eGFP (MOI 10) under different conditions (0.1% DMSO control, CPXV012 and control peptide UL49.5 in 0.1% DMSO). At 20 h.p.i., infection was assessed by fluorescence microscopy (bar size 1 mm). Results are representative of at least three independent experiments. BF: Bright field. GFP: Green fluorescent protein (MVA infection). (**B**) MJS cells were infected with MVA-eGFP (MOI 10) in the presence of 0.1% DMSO vehicle control, or CPXV012 peptide, or control peptide, both in 0.1% DMSO. At 18 to 20 h post infection, cells were harvested and analyzed for viral gene expression. The number of eGFP-positive cells was quantified using flow cytometry. Mean ± S.E.M. of three independent experiments is shown. (**C**) MJS cells were treated for 20 h with the indicated concentrations of CPXV012 or control peptide. Subsequently, cells were harvested and stained with DAPI for flow cytometric analysis. S.E.M. of three independent experiments is shown. (**D**) MJS cells infected with MVA-eGFP (MOI 10) in the presence of 100 µg/mL CPXV012 or UL49.5 (control) peptide in 0.1% DMSO or 0.1% DMSO only were lysed 20 h post infection. RNA was isolated and qPCR was performed for expression of viral genes B8R, H3L, and G8R. Mean ± S.E.M. of three independent experiments is shown. Log-transformed data were analyzed with one-way ANOVA followed by multiple comparisons Dunnett’s test (the mean of each column was compared to that of the DMSO control). (**E**) CPXV012 peptide inhibits infection with the poxviruses MVA-eGFP, VACV-eGFP, and CPXV-RFP/eGFP. MJS cells were infected with VACV-eGFP (MOI 10) or CPXV-RFP/eGFP (MOI 10) in the presence of 150 µg/mL CPXV012 or UL49.5 control peptide in 0.1% DMSO or 0.1% DMSO only. After 18 to 20 h, infection was quantified by cytometric analysis of fluorescent cells. Mean ± S.E.M. of three independent experiments is shown using different batches of peptide. Data were analyzed with one-way ANOVA followed by multiple comparisons Dunnett’s test (the mean of each column was compared to that of the DMSO control). (** *p* < 0.01; *** *p* < 0.001).

For qPCR analysis (Figure 1B), MJS cells were infected with MVA-eGFP (MOI 10) in the presence of 100 µg/mL of either CPXV012 peptide or control peptide in medium with 0.1% DMSO or in 0.1% DMSO only as vehicle control at 37 °C for 20 h. RNA was isolated using the RNeasy Mini Kit (Qiagen, Venlo, The Netherlands). A total of 3 μg of RNA was digested with 10 U of DNase I (Roche, Woerden, The Netherlands) and cDNA synthesis was performed by using 200 U Superscript III RNase H reverse transcriptase (Invitrogen, Amsterdam, The Netherlands), 7.5 pmol oligo(dT)_12–18_ (Invitrogen), 20 U of RNasin (Promega), and 10 mM each deoxynucleoside triphosphate (Qiagen). For semiquantitative analysis of viral mRNAs, cDNA was used as the template for a PCR with the indicated primers: B8R (fwd ATCCGCATTTCCAAAGAATG, rev ACATGTCACCGCGTTTGTAA), H3L (fwd GTCTTGAAGGCAATGCATGA, rev TCCCGATGATAGACCTCCAG) and G8R (fwd ATC GAT AAA CTG CGC CAA AT), and rev CTC CGC GGT AGA ACA CTG AT). Quantitative RT-PCR analysis was performed by using the LightCycler DNA Master SYBR green I kit (Roche) and LightCycler 1.5 (Roche). Gene expression was determined using the 2^−∆∆CT^ method. The results are quantified relative to the housekeeping gene GAPDH [27,28].

#### 2.4.2. HSV-1

MJS cells (10^5^/well) were seeded in a 24-well plate and cultured overnight at 37 °C. HSV-1-eGFP was mixed with CPXV012 peptide, control peptide UL49.5, or DMSO only in complete medium. DMSO concentration was 0.1% with or without peptide. The medium of cells was replaced with virus/peptide inoculum (MOI 0.1) and cells were incubated for 16 h at 37 °C. Cells were harvested, fixed using 1% formaldehyde, and the number of eGFP-positive cells was determined using flow cytometry.

#### 2.4.3. HBV

Confluent HepRG cells cultured in 48-well plates were differentiated for two weeks. Medium was removed and cells were left untreated, or CPXV012 peptide in medium containing 0.1% DMSO was added at the concentrations indicated. Medium containing 0.1% DMSO was used as vehicle control. Cells were infected with HBV (MOI 200) and incubated for 16 h. Medium was removed, cells were washed three times with PBS, fresh medium was added, and cultures were incubated for another 12 days with a medium change every three days. Thereafter, the supernatant was collected after centrifugation to remove cellular debris (5 min, 350× *g*) and HBeAg was quantified using commercial immunoassays as described [20]. HBV DNA was extracted from the supernatant using the BioSprint 96 One-For-All Vet Kit (Qiagen) and was quantified by qPCR.

#### 2.4.4. HIV-1

The EASY-HIT assay was performed as described previously [17]. Briefly, 1 × 10^4^ LC5-RIC cells were plated in 96-well plates and incubated at 37 °C for 24 h. Thereafter, cells were treated with medium containing 0.1% DMSO and the indicated concentrations of CPXV012 peptide or 0.1% DMSO only as vehicle control for 1 h prior to infection with HIV inoculum. After 48 h, cultures were assayed for cellular reporter gene expression by quantification of total fluorescence intensity of each culture using a fluorescence microplate reader (step 1). To assess titers of infectious virus in the culture supernatant (virion production in primary infected cells), 20 µL of medium was added to fresh LC5-RIC cells and incubated for another 72 h before reporter gene expression was quantified (step 2).

#### 2.4.5. Measles and VSV

Vero cells (1 × 10^5^ cells/well) were plated in 12-well plates and left untreated or treated with medium containing 0.1% DMSO and the indicated concentrations of CPXV012 peptide or 0.1% DMSO only as vehicle control. Cells were infected with MV-eGFP (MOI 0.1). Once maximum giant cell formation was observed at 48 h post infection, microscopic fluorescence images were taken by using an inverted microscope CKX41 (Olympus) with an LCachN/10×/0.40 Phc/1/FN22 UIS objective. Thereafter, the medium was removed, and infection was measured based on the expression of eGFP. Fluorescence was detected using an Infinite 200 PRO Tecan reader (fluorescence bottom reading for cell-based assays). For VSV infections, Huh7.5 cells (2 × 105 cells/well) were seeded in 12-well plates, and peptides were added at the concentrations indicated. DMSO was used as vehicle control. Cells were infected with VSVdeltaG (Luc) (MOI 0.6). At 18 h post infection, luciferase was measured using the luciferase assay system (Promega) and normalized to the protein content of the individual sample as determined by a Bradford assay.

#### 2.4.6. Adenovirus

HEK-293 cells (4 × 10^5^ cells/well) were seeded in 12-well plates in a total volume of 1 mL and incubated overnight at 37 °C. Cells were treated with medium containing 0.1% DMSO and the indicated concentrations of CPXV012 peptide or 0.1% DMSO only as vehicle control at 37 °C for 1 h. Afterward, cells were infected with an MOI of 10 with AdGOva for 24 h. Photometric analysis was performed with the Infinite 200 PRO Tecan to quantify the percentage of infected cells based on GFP-expression.

#### 2.4.7. Rift Valley Fever Virus

MJS cells (2 × 10^4^ cells/well) were seeded in a 96-well plate and incubated for two days at 37 °C. Cells were treated with medium containing 0.1% DMSO and the indicated concentrations of either CPXV012 peptide or control peptide UL49.5 or 0.1% DMSO only as vehicle control. After 15 min, RVFV-eGFP was added to the cells. Twenty-four hours after infection, cells were harvested and the percentage of eGFP-positive cells was determined using flow cytometry.

#### 2.4.8. Coxsackievirus B3

MJS cells (10^4^ cells/well) were seeded in a 96-well plate. After overnight incubation at 37 °C, cells were infected with RLuc-CVB3 in the presence of medium containing 0.1% DMSO and the indicated concentrations of CPXV012 peptide or 0.1% DMSO only as vehicle control. After 7 h of infection, cells were lysed and renilla luciferase expression levels were quantified using the Renilla Luciferase Assay System kit (Promega).

### 2.5. Preparation of Large Unilamellar Vesicles (LUVs)

Calcein-encapsulated LUVs were prepared using 1,2-dioleoyl-*sn*-glycero-3-phosphocholine (DOPC) or a mixture of DOPC and 1,2-dioleoyl-sn-glycero-3-phospho-L-serine (DOPS) (Avanti Polar Lipids) in a 7:3 molar ratio. A stock solution of DOPC and DOPS in chloroform (10 mM) were mixed in a glass tube. The solvent was evaporated with dry nitrogen gas yielding a lipid film that was subsequently kept in a vacuum desiccator for 20 min. Lipid films were hydrated for 30 min in buffer containing 10 mM Tris, 50 mM NaCl at pH 7.4 resulting in a total lipid concentration of 10 mM. For calcein-encapsulated LUVs, 50 mM of calcein was added during hydration. The lipid suspension was freeze-thawed ten cycles, at temperatures of −80 and +40 °C, respectively, and eventually extruded 10 times through 0.2 μM-pore size filters (Anotop 10, Whatman, UK). For the preparation of calcein-encapsulated LUVs, free calcein was separated from calcein-filled LUVs using size exclusion column chromatography (Sephadex G-50 fine) and eluted with 10 mM Tris-HCl, 150 mM NaCl buffer at pH 7.4. Finally, the phospholipid content of lipid stock solutions and vesicle preparations was determined as inorganic phosphate according to Rouser [29].

### 2.6. Circular Dichroism

Circular dichroism (CD) experiments were performed as described previously (15). Briefly, the CD spectra of 625 μM LUVs and/or 100 μg/mL peptides diluted in 10 mM MES buffer (pH 6.2) were recorded on a Jasco 810 spectropolarimeter (Jasco, Easton, MD) over a wavelength range of 200 to 250 nm. Each reported spectrum is the average of five independent scans recorded every 1 nm at a scan rate of 20 nm/min at room temperature in cuvettes with a path length of 1.0 mm.

### 2.7. Langmuir Monolayers

Peptide-induced changes in the surface pressure of a monomolecular layer (monolayer) of phospholipids at a constant surface area were measured using a Langmuir Microtrough XL device (Kibron, Helsinki, Finland). A Teflon trough was filled with 16 mL PBS (pH 7.4) and lipid monolayers of DOPC or DOPC/DOPS (ratio 7:3) were spread from a 0.5 mM stock solution in chloroform at the air–buffer interface. The buffer below the lipid monolayer (subphase) was continuously stirred using a magnetic stirrer. Upon stabilization of the initial surface pressure to 25 mN/m, a freshly prepared stock of peptide in DMSO was injected into the subphase, resulting in a final peptide concentration of 0.25 μM.

### 2.8. Membrane Permeability Assay

Membrane permeability was measured in standard 96-well transparent microtiter plates in a plate reader (Spectrafluor, Tecan, Salzburg, Austria). Peptides (5 μL of 0.2 mM in DMSO) were added to calcein-containing LUVs (195 μL of lipid vesicles (50 μM) in 10 mM Tris–HCl, 100 mM NaCl buffer (pH 7.4)). As positive control human islet amyloid polypeptide (hIAPP; Bachem) 5 μL of a 0.2 mM in DMSO) was added to calcein-containing LUVs. As a negative control, murine IAPP ((mIAPP; Bachem) 5 μL of 0.2 mM in DMSO) was added to calcein-containing LUVs. For blank only, 5 μL DMSO was added to calcein-containing LUVs. Directly after the addition of all components, the microtiter plate was shaken for 10 s. Fluorescence was measured from the top, every 5 min, using a 485 nm excitation filter and a 535 nm emission filter at 25 °C. The maximum leakage at the end of each measurement was determined by adding 1 μL of 10% Triton X-100 to a final concentration of 0.05% (*v/v*). The release of fluorescent dye was calculated according to Equation (1):

L(t) = (Ft − F0)/(F100 − F0)(1)

L(t) is the fraction of dye released (normalized to membrane leakage), Ft is the measured fluorescence intensity, and F0 and F100 are the fluorescence intensities at times t = 0 and after addition of Triton X-100, respectively. All membrane leakage assays were performed two times, each in duplicate, on different days, using different IAPP stock solutions.

### 2.9. Statistical Analysis

Statistical significance was analyzed by one-way ANOVA testing, followed by Dunnett’s multiple comparisons test (the mean of each column was compared to that of the DMSO control), using GraphPad Prism 8.0.1. In Figure 1D, statistical analysis was performed on the plotted data transformed as follows: Y = Log(Y). *p*-values of <0.05 were considered significant (* *p* < 0.05; ** *p* < 0.01, *** *p* < 0.001).

## 3. Results

### 3.1. CPXV012 Peptide Prevents Infection by Different Viruses in Cell Culture

We tested a number of peptides for their ability to inhibit TAP and subsequent MHC-I antigen presentation. These peptide sequences were based on the active domains of viral TAP-inhibiting proteins CPXV012 (from cowpox virus) and UL49.5 (from pseudorabies virus) [30]. While testing their ability to block MHC I antigen presentation during virus infection, we found that the CPXV012 peptide was uniquely capable of inhibiting virus reporter expression. To assess the effect of CPXV012 peptide on viral infection, different infection inhibition assays were performed using a variety of viruses and cell types. Cells were treated with CPXV012 peptide (34 amino acids, QEGISRFKICPYHWYKQHMSLLFRRYYHKLDSII) or a similar-length control peptide derived from the pseudorabies virus protein UL49.5. The viral inoculum was mixed with the peptide at different concentrations and immediately added to cells. After incubation for the indicated times, infection was determined based on viral gene expression, or viral DNA or mRNA synthesis.

Infection of the human melanoma cell line MelJuSo (MJS) with recombinant modified vaccinia virus Ankara (MVA) expressing eGFP (MVA-eGFP) was confirmed via microscopy (Figure 1A). The cell monolayer showed the typical cytopathic effect upon infection (in the presence of DMSO, peptide vehicle control; bright field) and eGFP fluorescence could be detected. A similar pattern was verified in the presence of the control peptide. In contrast, upon infection and incubation with the CPXV012 peptide (100 µg/mL), the monolayer was microscopically indiscernible from that of the uninfected control. MVA-eGFP infection was inhibited in a concentration-dependent manner (Figure 1B). Inhibition starts at 50 µg/mL and is stronger upon increasing peptide concentrations, reaching 98.3% (± 0.3) at 150 µg/mL CPXV012 peptide (Figure 1B). To test potential cytotoxicity of the peptides, the amount of DAPI-negative (live, i.e., cells whose membrane was not compromised/permeable) cells was quantified. We did not observe any decrease in live cells up to the highest concentrations (200 µg/mL) (Figure 1C). To further test any effect on cell viability, other assays were performed. The WST-1 assay measures the net metabolic activity of the cells (it is based on the enzymatic conversion of the WST-1 salt into the colored dye formazan in viable cells), and the Neutral Red uptake assay relies on the staining of lysosomes in viable cells upon active transport of the cationic dye. MJS cell viability was not severely impaired upon addition of up to 200 µg/mL CPXV012 peptide using the WST-1 assay (Figure S1A), and the Neutral Red assay (Figure S1B). Cell titer blue assays were performed to test the viability of other cell lines (Vero and Huh7.5 cells) used in this study in the setting of other viral infections. Again, no significant effect was observed at the highest peptide concentrations (Figure S1C).

To confirm the inhibitory effect of CPXV012 peptide on infection of MJS with MVA-eGFP, qPCR analysis for expression of viral genes was performed (Figure 1D). CPXV012 peptide decreased the expression of the early-expressed gene B8R up to two logs, relative to its expression upon infection in the presence of DMSO vehicle only. The decrease in expression of the intermediate-expressed gene G8R and late expressed gene H3L was more pronounced (more than two logs). No effect was seen if cells were treated with the control peptide compared to the DMSO sample (Figure 1D). To assess whether the inhibitory effect on MVA-eGFP infection also occurs in other cell types, HEK-293T, BHK21, and HeLa cells were used (Figure S2). In all cell lines, a strong decrease in infection (87.4 to 94.2%) was observed.

To investigate whether other poxviruses besides MVA were inhibited by CPXV012 peptide, we tested the effect on Vaccinia virus (VACV) strain WR and cowpox virus strain BR (CPXV). Again, infection of MJS with both viruses was inhibited (93 ± 1.7% for VACV and 72 ± 1.1% for CPXV) by CPXV012 peptide (150 µg/mL) while no effect was seen with the control peptide in comparison to the DMSO sample (Figure 1E). Thus, CPXV012 peptide inhibits infection of different members of the poxvirus family.

**Figure 2:**
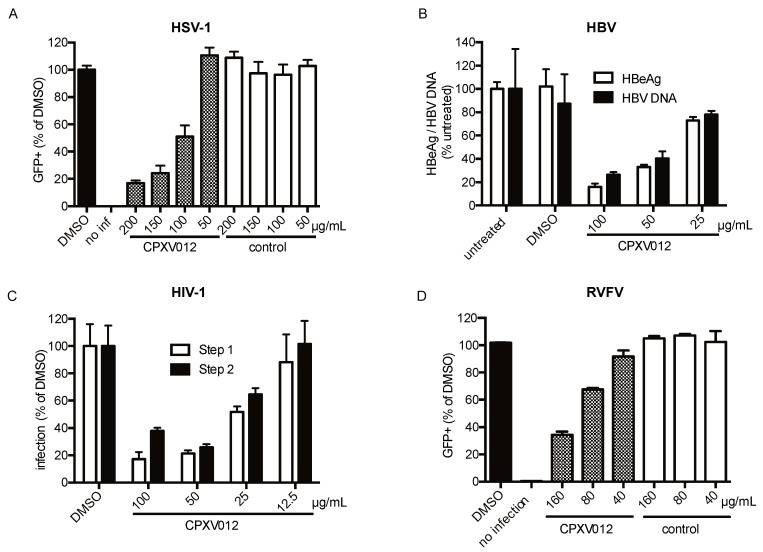
CPXV012 peptide inhibits infection with HSV-1, HBV, HIV, and RVFV. (**A**) CPXV012 peptide inhibits HSV-1 infection. MJS cells were left uninfected or infected with HSV-1 (MOI 0.1) in the presence of CPXV012 or UL49.5 as control at the indicated peptide concentrations in 0.1% DMSO or 0.1% DMSO only. At 16 h after infection, cells were harvested, and the amount of eGFP-positive cells was quantified using flow cytometry. Mean ± S.E.M. of three independent experiments is shown. (**B**) Effect of CPXV012 peptide on HBV infection. Differentiated HepRG cells were left untreated or CPXV012 peptide in 0.1% DMSO was added using the concentrations indicated or 0.1% DMSO only as control. Cells were infected with HBV (MOI 200) for 16 h. Subsequently, cells were washed and fresh medium was added. After 12 days, the supernatant was analyzed for HBeAg and HBV DNA. Mean ± S.E.M. of three independent experiments is shown. (**C**) CPXV012 peptide inhibits HIV infection. LC5-RIC cells were treated with the indicated concentrations of CPXV012 peptide in 0.1% DMSO or 0.1% DMSO only as control for 1 h and infected with HIV for 48 h according to the EASY-HIT assay system (17). Cellular reporter expression was quantified using a fluorescence microplate reader to assess the ability of HIV to infect LC5-RIC cells (Step 1). Next, 20 µL of culture supernatant was added to fresh LC5-RIC cells and incubated for another 72 h before fluorescence detection was performed to assess virion production from the first round of infection (Step 2). Data are shown as the percentage of infection referred to the respective DMSO control which was set to 100%. Mean ± S.E.M. of three independent experiments is shown. (**D**) CPXV012 peptide inhibits infection with RVFV. MJS cells were left uninfected or infected with RVFV-eGFP in the presence of CPXV012 or control peptide (UL49.5) at the indicated peptide concentrations in 0.1% DMSO or 0.1% DMSO only. After 24 h, cells were harvested and the amount of eGFP-positive cells was quantified using flow cytometry. Mean ± S.E.M. of three independent experiments is shown.

**Figure 3:**
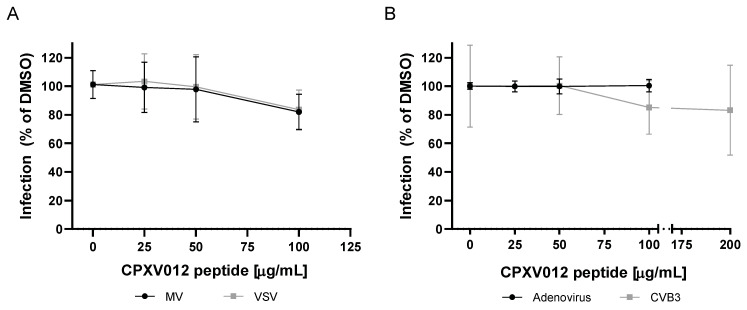
CPXV012 peptide does not inhibit infection with Measles virus, VSV, Adenovirus, or Coxsackie B3 virus and no difference was observed with the control peptide UL49.5 (data not shown). (**A**). Measles virus infection: Vero cells were treated with the indicated concentrations of CPXV012 peptide or DMSO as control, and infected with eGFP-expressing measles virus (MV-eGFP) (MOI 0.1). Once maximum giant cell formation was observed (approximately 48 h post infection), infection was quantified by detection of eGFP using a microplate reader. VSV infection: Huh7.5 cells were treated with the indicated concentrations of CPXV012 peptide in 0.1% DMSO or 0.1% DMSO only, and infected with luciferase-expressing VSV-deltaG (Luc) (MOI 0.6). At 18 h post infection, luciferase activity was measured to assess VSV infection of the culture. Data are shown as percentage of infection referred to the respective DMSO control which was set to 100%. Mean ± S.E.M. of each three independent experiments is shown. (**B**) Adenovirus infection: HEK-293 cells were treated with the indicated concentrations of CPXV012 peptide or DMSO for 1 h and infected with eGFP-expressing adenovirus (AdGOva) (MOI 10) for 24 h. Infection was quantified by cytometric analysis of eGFP expression. Coxsackievirus B3 infection: MJS cells were infected with RLuc-CVB3 in the presence of the indicated concentration of peptide or DMSO as vehicle control. After 7 h, cells were lysed and Renilla Luciferase expression was quantified. Data are shown as percentage of infection referred to the respective DMSO control which was set to 100%. Mean ± S.E.M. of each three independent experiments are shown.

To further characterize the inhibitory effect of CPXV012, in vitro infection inhibition assays were performed with a collection of viruses that differ in genome content (RNA or DNA) and structural composition (enveloped or non-enveloped). These viruses include herpes simplex virus-1 (HSV-1), Hepatitis B virus (HBV), HIV-1, adenovirus, measles virus, vesicular stomatitis virus (VSV), coxsackievirus B3 (CVB3), and Rift Valley fever virus (RVFV) (Table S1, Figure 2 and Figure 3).

The inhibitory effect of CPXV012 peptide on HSV-1-eGFP, a large dsDNA virus from the herpesvirus family, was evaluated upon infection of MJS cells. The cells were infected with HSV-1-eGFP in the presence of peptide or DMSO vehicle control. After 16 h, the amount of eGFP-positive cells was determined using flow cytometry (Figure 2A). As observed for the poxviruses, CPXV012 peptide showed a dose-dependent inhibition of HSV-1 infection (Figure 2A; 75.9 ± 5.7% when using 150 µg/mL peptide).

For HBV, an enveloped dsDNA virus of the hepadnavirus family, infectivity was monitored by quantifying the amount of viral envelope protein (HBeAg) and viral DNA in the supernatant of HepRG-infected cells (Figure 2B). Both HBeAg and viral DNA were decreased in the supernatant of cells treated with CPXV012 peptide in a concentration-dependent manner. At a peptide concentration of 100 µg/mL, a decrease of 84.0 ± 3.0% of HBeAg and 73.6 ± 2.3% of viral DNA was observed.

The infectivity of HIV-1, an enveloped ssRNA virus, was monitored using the EASY-HIT assay on LC5-RIC reporter cells. In this essay, both early and late phases of HIV replication are assessed: in step 1, the levels of a fluorescent reporter protein induced during the early phase of HIV replication are quantified, and in step 2 the production of infectious virions in primary infected cells is determined. HIV infection was reduced in the presence of CPXV012 peptide, but not in the presence of DMSO vehicle control (Figure 2C). Both primary viral infection (step 1) and viral replication (step 2) were dose-dependently affected, with an inhibition of 82.7 ± 4.9% (step 1) and 62.2 ± 2.4% (step 2) at the highest peptide concentration (100 µg/mL).

For RVFV, an enveloped ssRNA virus of the Bunyavirus family, infection was monitored by viral eGFP expression 24 h post infection (Figure 2D). Infection was reduced by CPXV012 peptide in a concentration-dependent manner (65.5 ± 2.3% at 160 µg/mL).

In contrast, CPXV012 peptide showed no effect on infection by the measles virus, an enveloped ssRNA virus of the paramyxovirus family (Figure 3A). The formation of measles virus-induced syncytia was unchanged (Figure S3). Replication of measles virus and cell-to-cell spread was assessed by quantification of viral titers present in infected cultures using plaque assays. No difference was found between CPXV012 peptide-treated samples, the DMSO vehicle control, or the control peptide (data not shown). For VSV, an enveloped ssRNA virus of the rhabdovirus family, virus-encoded Renilla luciferase activity was measured 18 h after infection (Figure 3A). Luciferase activity was unchanged in the presence of CPXV012 peptide.

Next, the effect of CPXV012 on the infection of two non-enveloped viruses was tested. The infectivity of adenovirus, a dsDNA virus, was not altered in the presence of CPXV012 peptide, as measured by viral eGFP expression (Figure 3B). For the coxsackie B3 virus, a non-enveloped ssRNA virus, infection was monitored by the activity of the virus-encoded Renilla luciferase. No change in Renilla luciferase activity was observed in the presence of CPXV012 or control peptide (Figure 3B).

These results identify the CPXV012 peptide as an antiviral peptide acting on enveloped viruses (summarized in Table S1).

### 3.2. CPXV012 May Bind to Viral Particles

**Figure 4:**
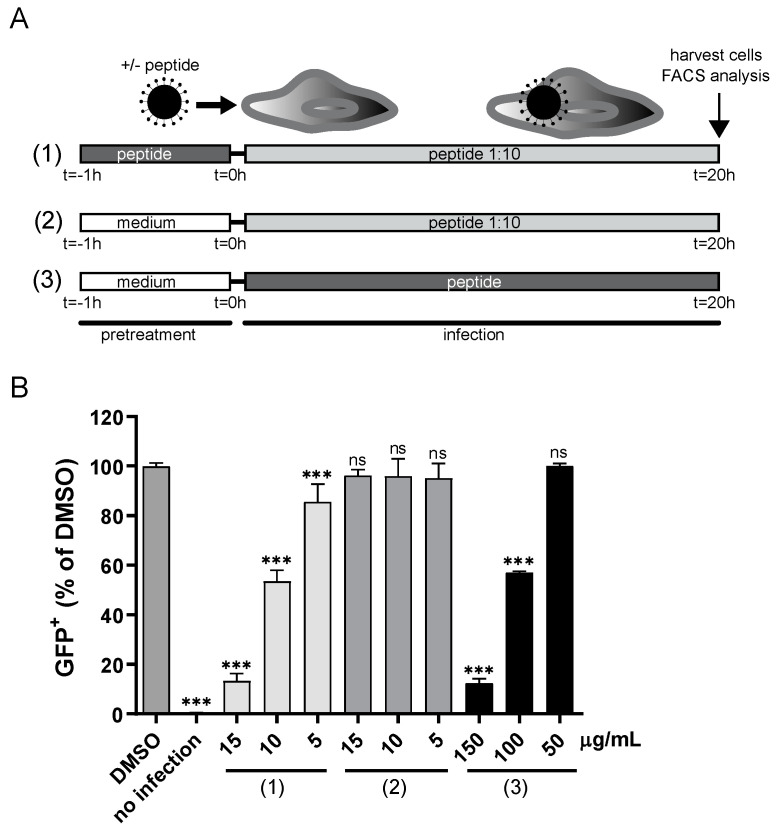
CPXV012 peptide inhibits infection by binding to viral particles. (**A**) Timeline of infection experiment as shown in (**B**). (1) MVA-eGFP was pretreated with 50, 100, or 150 µg/mL peptide or DMSO in culture medium at 37 °C for 1 h. Subsequently, the virus-peptide pre-incubation mixture was diluted ten times and used to infect MJS cells (corresponding to an MOI of 10), resulting in a final concentration of 5, 10, and 15 µg/mL peptide in the culture medium. (2–3) MVA-eGFP was incubated in culture medium only at 37 °C for 1 h. The virus mixture was added to MJS cells in the presence of 5, 10, or 15 µg/mL peptide (2) or 50, 100, or 150 µg/mL peptide (3) in the culture medium. (**B**) After 18 to 20 h of infection as shown in (**A**), the amount of infected eGFP-positive cells was quantified using flow cytometry. Mean ± S.E.M. of three independent experiments is shown using different batches of peptide. Data were analyzed with one-way ANOVA followed by multiple comparisons Dunnett’s test (the mean of each column was compared to that of the DMSO control). (*** *p* < 0.001).

To find out whether CPXV012 peptide mediates its inhibitory effect by interacting directly with viral particles, we performed a modified inhibition assay using MJS cells and MVA-eGFP. Instead of simultaneously adding CPXV012 peptide and virus to the cells, viral particles were pretreated with CPXV012 peptide for 1 h at 37 °C. After this preincubation, the virus-CPXV012 peptide mixture was diluted ten times and used to infect cells. Thus, the final peptide concentration during infection was 5 to 15 µg/mL (Figure 4A). Pretreating the virus with a high concentration of CPXV012 peptide before infection affected infectivity to a similar level as the presence of a high concentration of CPXV012 peptide during infection (Figure 4B). This effect was not due to the remaining CPXV012 peptide in the culture medium, as the diluted peptide alone was not sufficient to block infection when added directly to the cells. These data suggest that the peptide can directly act on viral particles.

### 3.3. CPXV012 Peptide Variants Differentially Affect Virus Infection

**Figure 5:**
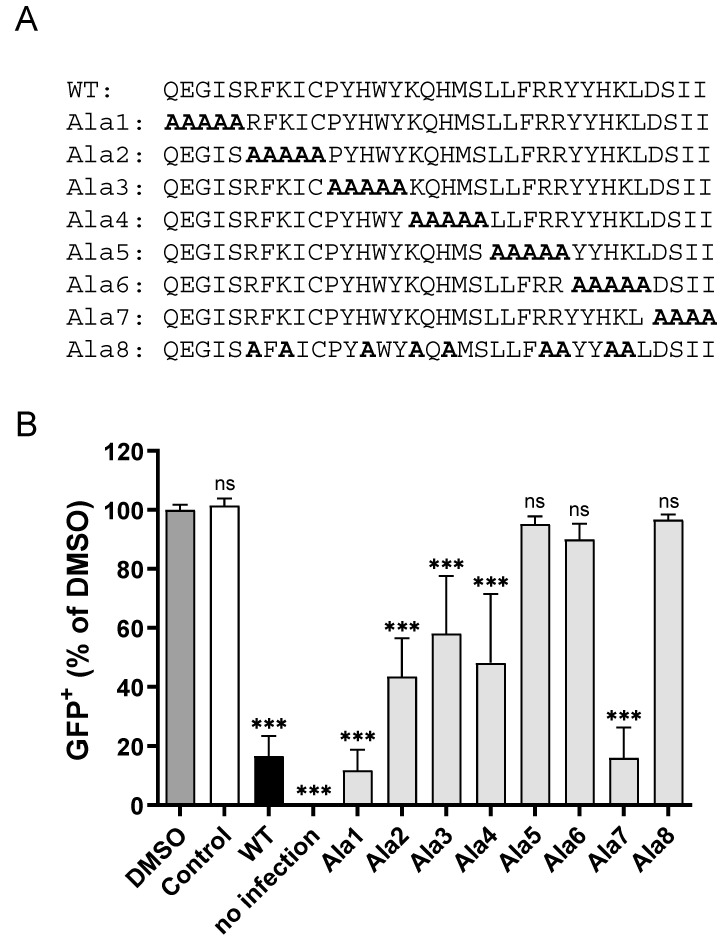
CPXV012 peptide variants differentially affect virus infection. (**A**) Amino acid sequence of CPXV012 peptide variants used in the experiment (**B**). (**B**) MJS cells were infected with MVA-eGFP at an MOI of 20 in the presence of 100 µg/mL of the peptide indicated or DMSO as vehicle control. After 20 h, cells were harvested, and the amount of eGFP-positive cells was quantified using flow cytometry. Mean ± S.E.M. of three independent experiments is shown using different batches of peptide. Data were analyzed with one-way ANOVA followed by multiple comparisons Dunnett’s test (the mean of each column was compared to that of the DMSO control). (*** *p* < 0.001).

To determine the amino acid residues within the CPXV012 peptide crucial for virus inhibition, alanine substitution variants of the CPXV012 peptide were synthesized. These variants had small stretches of amino acid residues replaced by alanine residues (Figure 5A). To test the inhibitory capacity of these variants, MJS cells were infected with MVA-eGFP in the presence of 100 µg/mL of each of these peptides (Figure 5B). CPXV012 variants with the N-terminal five amino acid residues (Ala1) or the C-terminal four amino acid residues substituted by alanine (Ala7) inhibited MVA infection to similar levels as wild-type CPXV012 peptide. Substituting amino acid residues 6 to 20 for alanine (CPXV012-Ala2/Ala3/Ala4) significantly affected the inhibitory capacity of the peptides. Substituting amino acid residues 20 to 30 for alanine (Ala5 and Ala6) completely abolished the inhibitory effect of CPXV012 peptide. Interestingly, the CPXV012 peptide variants with lower or no inhibitory capacity also had a lower net positive charge at pH 7.4 compared to the wild type peptide, due to the substitution of the charged amino acid residues lysine, arginine, or histidine, the latter being only partially charged. To test the role of these amino acid residues in virus inhibition, a peptide was synthesized with the charged amino acid residues replaced for alanine (Ala8). Indeed, this CPXV012 peptide variant lost its inhibitory capacity, thus confirming the role of basic amino acid residues in inhibiting virus infection.

### 3.4. CPXV012 Peptide Interacts with Charged Phospholipids

**Figure 6:**
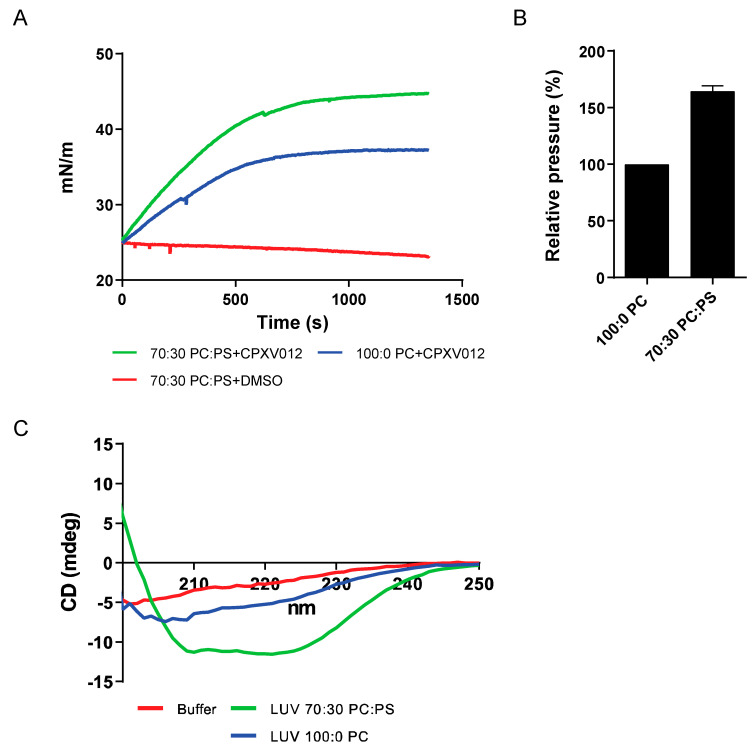
CPXV012 peptide interacts with PS. (**A**) CPXV012 peptide preferentially integrates into lipid monolayers composed of PC:PS (70:30). Langmuir monolayers of PC or PC:PS (70:30) with an initial surface pressure of 25 mN/m were formed over an aqueous subphase. Peptide was injected into the subphase at t = 0 s. As vehicle control, DMSO was injected in the subphase of PC:PS (70:30) monolayers. Results are representative of three independent experiments. (**B**) Quantification of CPXV012 peptide integration into lipid monolayers composed of PC:PS (70:30), as compared to monolayers composed of PC only (set at 100%). Mean ± S.E.M. of three independent experiments is shown. (**C**) CPXV012 peptide adopts different secondary structures depending on the presence of PS. CD spectrum of CPXV012 peptide was determined in the presence of aqueous buffer or LUVs composed of PC or PC:PS (70:30). Results are representative of three independent experiments.

The preferential inhibition of enveloped viruses suggests that CPXV012 peptide interacts with a common structure within these viral particles. A major constituent of these virions is the phospholipids forming the lipid bilayer of the viral envelope. To test the interaction between CPXV012 peptide and phospholipids, Langmuir monolayers were formed using the zwitterionic phospholipid phosphatidylcholine (PC) and the anionic phosphatidylserine (PS) on an aqueous buffer. Upon stabilization of the monolayer at an initial surface pressure of 25 mN/m, CPXV012 peptide was injected into the monolayer aqueous subphase and the surface pressure was measured for ~25 min (Figure 6A). A change in surface pressure is interpreted as the integration of CPXV012 peptide into the lipid monolayer. Although the surface pressure of monolayers formed by the zwitterionic PC changed rapidly upon the addition of CPXV012 peptide, the shift in surface pressure was much higher for monolayers formed by a 7:3 mixture of PC and the negatively charged PS (Figure 6B). These data suggest a preferred interaction between CPXV012 and the anionic PS. This is further supported by the circular dichroism (CD) experiments used to determine the secondary structure of CPXV012 peptide (Figure 6C). The CD spectrum of CPXV012 was measured in the presence of large unilamellar vesicles (LUVs), lipid vesicles consisting of PC only, or of PC and PS. In aqueous MES buffer or in the presence of PC, CD spectra of CPXV012 peptide were rather similar and becoming more negative at a shorter wavelength, suggesting the presence of a significant amount of random coil structure. However, upon addition of PS to PC lipids using a similar ratio as for the monolayer experiments, the CPXV012 peptide acquired a different CD spectrum with minima around 208 and 222 nm, typical for an alpha-helical structure. Thus, CPXV012 peptide adopts different secondary structures, depending on the presence of PS.

**Figure 7:**
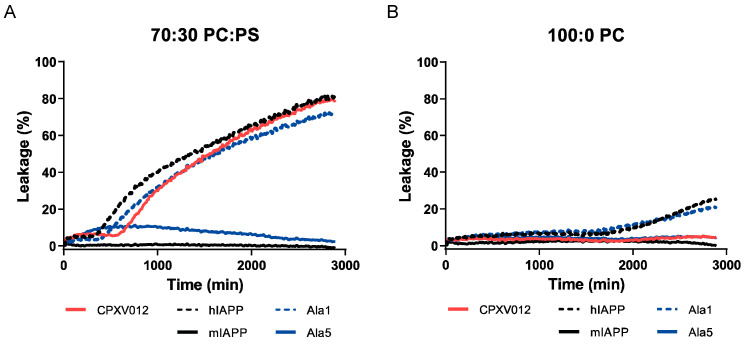
CPXV012 peptide disrupts membranes composed of PS. Calcein-containing PC:PS (7:3) LUVs (**A**) or PC LUVs (**B**) were incubated with human IAPP (hIAPP) as the positive control, murine IAPP (mIAPP) as the negative control, CPXV012 peptide, CPXV012 peptide variants Ala1 or Ala5 (see Figure 5A for amino acid sequences). Calcein leakage from the vesicle lumen was quantified using a microplate reader. Membrane leakage was compared to fully disintegrated detergent-treated LUVs (set at 100%). Results are representative of three independent experiments.

To gain further insight into the interaction between CPXV012 peptide and lipid membranes, the effect of the peptide on the integrity of lipid membrane vesicles was evaluated by membrane leakage assays using LUVs. These LUVs were composed of PC:PS and contained the self-quenching fluorophore calcein in their lumen. As soon as vesicle integrity is compromised, calcein leakage induces an increase in fluorescence, which can be measured in the supernatant using a fluorometer. As described previously, addition of human IAPP, but not murine IAPP, induced leakage of LUVs composed of PC and PS, whereas LUVs composed of PC alone were more resistant to leakage by human IAPP (Figure 7A,B) [31]. The addition of 25 µg/mL CPXV012 peptide disrupted the LUV membranes composed of both PC and PS, whereas membranes containing PC alone were unaffected. As described for human IAPP [32], membrane leakage by CPXV012 peptide is not instant but is preceded by a lag phase of several hours.

Two alanine substitution peptides, Ala1 and Ala5 (see Figure 5), were also tested. CPXV012 peptide variant Ala1, which inhibited virus infection, induced membrane leakage of PC:PS LUVs to similar levels and kinetics as wt CPXV012 peptide. In contrast to CPXV012, this variant was able to induce slight leakage of LUVs composed of PC only. Ala5, which had no effect on virus infection, did not compromise the integrity of either one of the LUV membranes (Figure 7B).

In conclusion, the capacity of CPXV012 peptide to inhibit virus infections correlates with its ability to disrupt lipid membranes that contain PS.

## 4. Discussion

In this study, we identified the CPXV012 peptide as a broadly-acting antiviral agent that probably interacts with the anionic phospholipid PS. The antiviral potency of the CPXV012 peptide can be considered modest, yet we believe that this peptide may be a candidate for biochemical improvement. The interaction between the CPXV012 peptide and PS is likely mediated by opposing electrostatic forces determined by cationic residues within the peptide and the anionic head groups of PS. In addition, hydrophobic and aromatic amino acid residues (which make up more than 30% of CPXV012 peptide) may facilitate penetration of the peptide into the lipid phase of the membrane. As shown for other peptides, such membrane interactions may promote the formation of an alpha-helical secondary structure of otherwise unstructured peptides [33]. Due to the distribution of charged and hydrophobic residues over the length of the CPXV012 peptide, the alpha-helix can be expected to have somewhat of an amphipathic character. This may allow it to bind with an orientation that is roughly parallel to the membrane surface [34] or to assemble into oligomeric transmembrane pores [35]. These biophysical properties of CPXV012 peptide are shared with many antimicrobial host defense peptides [36], that form a crucial innate immune barrier by targeting the negatively charged surfaces of bacteria and certain fungi. Like CPXV012 peptide, these small peptides (up to 50 residues) have a net positive charge and high affinity for anionic phospholipids. The structural rearrangements occurring upon membrane binding are crucial for the antimicrobial activity and can trigger pore formation, membrane depolarization, and disruption of the bacterial membrane [37,38].

Although less studied, antimicrobial peptides may also affect virus infection by different mechanisms. These mechanisms include interference with viral entry (binding to receptors, fusion), inhibition of viral replication, reduction/suppression of viral gene expression, immunomodulation, viral aggregation, and disruption of the integrity of the viral membrane (reviewed in [6,7]). While a list of peptides is reported to disrupt viral envelopes, their mode of action has not been formally tested. Still, a number of them were shown to directly affect viral membranes, although the specific interacting partners remain elusive. Examples include the human cathelicidin LL-37 and its murine equivalent mCRAMP, which disrupt the membranes of RSV, influenza A virus and VACV, and can prevent virus infection in vivo [39,40,41]. Although their effect on the infectivity of other viruses is not known, the structural similarities between these cationic peptides and CPXV012 peptide may suggest that these and other antimicrobial peptides possess a broader antiviral activity. Electron microscopy has also provided indications of envelope disruption with the cationic peptides Tachyplesin (VSV) [7], Temporin B (HSV-1) [42], and more recently Urumin (H1-bearing influenza A viruses) [43].

Membrane leakage induced by CPXV012 peptide was preceded by a lag phase, as was also observed for human IAPP. For human IAPP, this lag phase originates from the formation of transient oligomeric intermediates prior to the assembly of membrane-disruptive aggregates [32]. Likewise, disruption of membranes by CPXV012 peptides may be linked to the formation of (transient) oligomeric structures. The shorter lag time in the presence of PS is possibly due to more efficient binding of the peptide to the membrane: upon favorable electrostatic interactions, CPXV012 peptides achieve a higher local concentration and therefore the assembly process is supported. The lag phase of several hours appears to be inconsistent with the almost immediate effect observed in virus infection assays. There can be several reasons for this apparent discrepancy. First, the local peptide concentration at the membrane surface in the virus assays is likely to be much higher than in the leakage experiments, facilitating the oligomerization process. Second, antiviral activity may not require the formation of pores that are large enough to allow passage of calcein. Instead, binding to the viral membrane and/or insertion of hydrophobic amino acid side chains between the lipid acyl chains by itself already may be sufficient for an antiviral effect. Third, other factors may be involved in the virus infection assays, such as interactions with viral (glycol)proteins in addition to PS, that help accelerate peptide oligomerization on the viral surface.

Our data indicate that the CPXV012 peptide neutralizes the infectivity of viruses from diverse families. In vitro assays indicate that the CPXV012 peptide directly interacts with membranes. The disruptive behavior of the CPXV012 peptide on artificial membrane vesicles suggests that the CPXV012 peptide may similarly affect viral membranes. As viral particles do not have a membrane repair mechanism, this damaging effect may particularly destabilize and disrupt viral particles. Extensive membrane damage may also hamper virus fusion to the host cell membrane by impacting the fluidity and curvatures of lipid membranes. Lipids including PS play an essential role in the induction of these membrane curvatures and subsequent membrane fusion [44,45]. Even in the absence of membrane damage, the interaction between CPXV012 peptide and PS in the viral envelope may interfere with virus infectivity by sterically blocking the interaction between viral PS and host cell PS receptors. A similar inhibitory mechanism was observed using the PS-specific antibody Bavituximab [3,46], or the PS-binding protein Annexin V [47,48]. CPXV012 peptide may also indirectly affect virus infection by binding to PS on the host cell membrane. Specific microdomains of the host cell plasma membrane are enriched for PS, including lipid rafts [49]. These sites are used by certain viruses as an entry point [50]. Although PS typically would reside in the inner leaflet of the plasma membrane, there are indications that some viruses induce redistribution of PS to the outer membrane to promote/facilitate entry [50]. In addition, some viruses use lipid rafts as budding sites when exiting the host cell [51].

The effect of CPXV012 peptide on the viruses used in this study and potential inhibitory mechanism(s) are discussed in more detail.

### 4.1. Poxviruses

The CPXV012 peptide was able to inhibit infection with different orthopoxviruses (MVA, VACV, and CPXV) in several cell lines, using various detection methods. Notably, early gene expression, which starts immediately after virus entry, was highly reduced and indicates a block at the entry step. Viral particles of the poxviridae family adopt distinct structures and composition of the outer membrane, such as mature virions (MVs, also named IMVs, intracellular mature virus) [52]. MVs represent the majority of infectious progeny; they are very stable and largely responsible for viral spread. In viral preparations, MVs are the most abundant virion form. Importantly, membranes of MVs have been shown to contain large amounts of PS that is essential for entry [47,53]. We found that CPXV012 peptide binds to PS and thus likely mediates its inhibitory effect on poxvirus infection by interacting with PS in the viral membrane. This was confirmed by the observation that pretreating virions with CPXV012 peptide effectively blocked infection.

### 4.2. HSV-1

Inhibition of HSV-1 by cationic peptides has been previously reported, including the antimicrobial peptide LL-37 that presumably acts by targeting factors within the viral membrane [54,55,56]. It is believed that HSV-1 derives its final envelope from membranes of the late secretory pathway (e.g., trans-Golgi network) and/or endosomal pathway [57]. The inhibition of HSV-1 by CPXV012 peptide suggests that PS in the HSV-1 envelope is a potent target for antiviral therapy.

### 4.3. HBV

CPXV012 peptide affected HBV infection as measured by a decrease in viral DNA and viral antigen in the supernatant of infected cultures. HBV particles are thought to acquire their envelope from multivesicular bodies [58], which are rich in PS [59]. The role of PS in HBV infection is underlined by studies showing that antibodies specific for PS block HBV infection [60]. Similar to poxviruses, PS may play a role in virus entry through apoptotic mimicry [61,62]. PS may also play a role in the proper folding of HBV envelope proteins [63].

### 4.4. HIV

The presence of PS in the membrane of HIV particles was shown to be an important cofactor for infection and blocking of PS led to a decrease in infection [64,65]. In addition, HIV infection was inhibited by the addition of lipid vesicles composed of PS that competed with the HIV particles for plasma membrane association, while lipid vesicles containing PC had no effect [64]. Therefore, the binding of CPXV012 peptide to PS in the viral envelope could explain the inhibitory effect observed in the EASY-HIT assay.

### 4.5. RVFV

The inhibition of RVFV infection by CPXV012 peptide suggests that PS is critical for RVFV infection. Although the lipid composition of RVFV is unknown, the viral envelope of the Uukuniemi virus, a related member of the bunyavirus family, is enriched for PS [66]. Like the Uukuniemi virus, RVFV obtains its membrane by budding into the Golgi membrane, suggesting that PS may also be enriched in these virus particles [67,68].

### 4.6. Viruses Not Affected by CPXV012 Peptide

No effects of CPXV012 peptide were observed on infection with adenovirus, coxsackie B3, measles virus, and VSV. Although the measles virus and VSV are enveloped viruses, the role of PS during infection is limited for these viruses. For the measles virus, different phospholipids were tested for their ability to reassemble with the viral envelope glycoproteins and to restore hemolytic activity. In contrast to phosphatidylethanolamine and PC, PS completely failed to restore hemagglutination activity [69]. For VSV, early studies considered PS expressed on the host cell important for cell entry [70]. In contrast, a more recent study found no correlation between PS surface levels and VSV binding. Moreover, masking of phosphatidylserine with annexin V during infection did not affect VSV binding to cells, or entry of virus particles pseudotyped with VSV-G [64,71]. Thus, the limited role of PS during entry of VSV and measles virus may explain the lack of inhibition by CPXV012 peptide.

Both adenovirus and Coxsackievirus B3 are non-enveloped viruses and the nucleocapsid does not contain any phospholipids (nor glycoproteins) CPXV012 peptide could bind to. CPXV012 peptide binding to the host cell membrane might not be sufficient for the steric impediment of viral ligand–cellular receptor interaction. Supporting this, the adenoviral elongated fiber protein responsible for binding to the host cell is flexible and large in size (9 to 30 nm), as is the coxsackie and adenovirus receptor (CAR) on the host cell [72].

CAR is also important for the infection of free CVB3 particles [73]. In addition, CVB3 may use a PS-dependent entry strategy for the bulk transmission of virus particles [74]. Infected cells release clusters of virus particles wrapped in PS-enriched membranes. These vesicles enhance subsequent virus entry in a process that highly depends on the presence of PS [74]. As CPXV012 peptide had no effect on CVB3 virus infection, PS may not play a significant role in our study. This discrepancy likely results from our virus production protocol that involves repeated freeze-thawing to liberate virus particles. Although necessary to obtain high virus titers, this method also releases CVB3 from PS-enriched vesicles [74].

In conclusion, the CPXV012 peptide-mediated inhibition of virus infection correlates well with the observed interaction between CPXV012 peptide and PS. The broad inhibitory range may thereby be further extended to other viruses for which PS has a crucial function during the viral life cycle. These viruses include clinically and economically important pathogens such as Ebola virus, Lassa virus, dengue virus, and poliovirus [61].

The differences between viral and host membranes make PS an Achilles’ heel that can be targeted by the CPXV012 peptide. It will be worth investigating if other cationic peptides, including certain antimicrobial peptides, can also target PS within the viral envelope, and perhaps fulfill a similar function as broad-range antiviral agents. Analyses of the peptide’s biophysical and physicochemical properties can shed some light on how to improve the moderate efficiency of CPXV012 and other cationic peptides. Highly efficient antiviral peptides are interesting candidates for prophylactic treatment and antiviral therapy, as they do not require active replication and target viral components that are less likely to develop drug-resistance [75].

## Supplementary Materials

The following are available online at https://www.mdpi.com/2073-4409/9/9/1989/s1, Table S1: Inhibitory effect of CPXV012 peptide on enveloped and non-enveloped viruses of different families. Figure S1: CPXV012 peptide does not affect cell viability. Figure S2: CPXV012 peptide (CPX) prevents infection with MVA in different cell lines. Figure S3: CPXV012 peptide does not affect infection with the measles virus.
