# Supplementary material for: A Broad-Spectrum Antiviral Peptide Blocks Infection of Viruses by Binding to Phosphatidylserine in the Viral Envelope
Source: Cells. 2020 Aug 29;9(9):1989. doi: 10.3390/cells9091989 (PMC7563927; doi:10.3390/cells9091989)
Supplement: Supplementary file 1 [file cells-09-01989-s001.pdf]

Supplementary Materials

Table 1 - Inhibitory effect of CPXV012 peptide on enveloped and non-enveloped viruses of different families

Virus	Family	Genome	Envelope	Inhibition
MVA	Poxviridae	dsDNA	yes	yes
VACV-WR	Poxviridae	dsDNA	yes	yes
CPXV-BR	Poxviridae	dsDNA	yes	yes
HSV-1	Herpesviridae	dsDNA	yes	yes
HBV	Hepadnaviridae	dsDNA-RT	yes	yes
HIV-1	Retroviridae	ssRNA-RT	yes	yes
RVFV	Bunyaviridae	ssRNA	yes	yes
Measles	Paramyxoviridae	ssRNA	yes	no
VSV	Rhabdoviridae	ssRNA	yes	no
Adenovirus	Adenoviridae	dsDNA	no	no
CVB3	Picornaviridae	ssRNA	no	no

MVA: Modified Vaccinia virus Ankara; VACV-WR: vaccinia virus strain Western Reserve; CPXV-BR: cowpox virus strain Brighton Red; HSV-1: herpes simplex virus-1; HBV: Hepatitis B virus; RVFV: Rift Valley fever virus; VSV: vesicular stomatitis virus; CVB3: coxsackie virus B3.

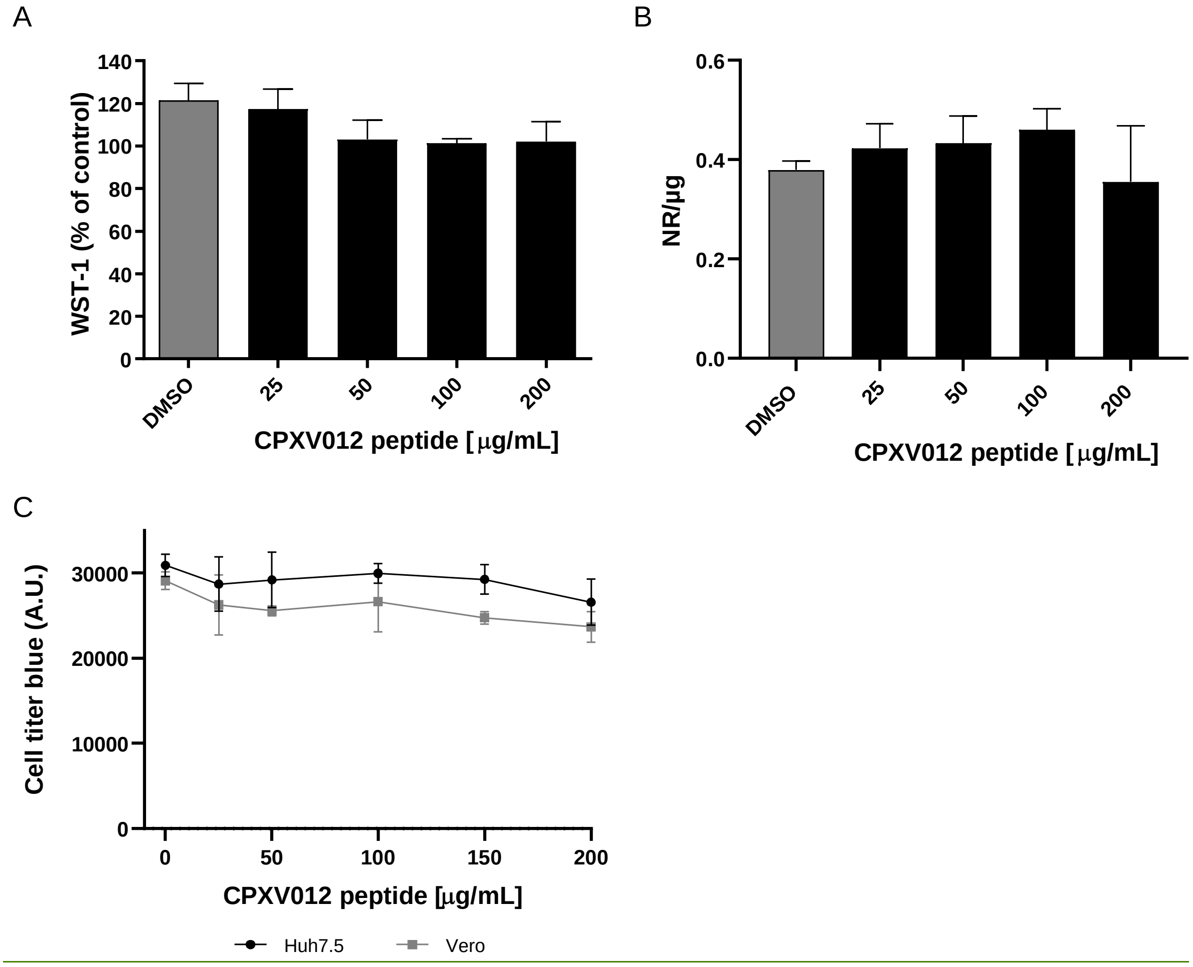

Figure S1: CPXV012 peptide does not affect cell viability. (A-C) Cells were treated with the indicated concentrations of peptide or DMSO as vehicle control. S.E.M. of three independent experiments is shown. (A) Viability of MJS cells was measured by WST-1 assay or (B) Neutral Red uptake. (C) Viability of Huh7.5 and Vero cells was measured by the cell titer blue assay. A.U.: arbitrary units. S.E.M. of three independent experiments is shown.

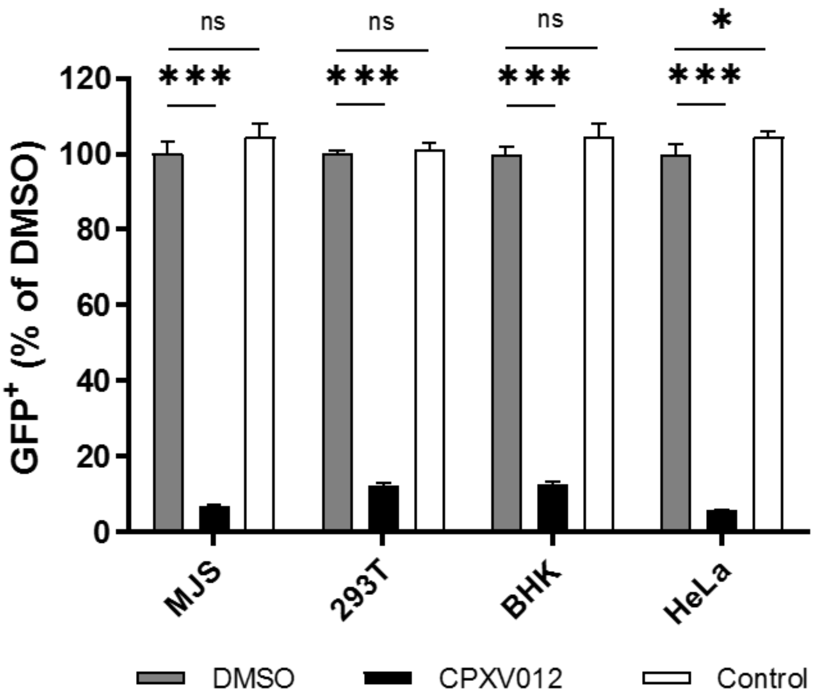

Figure S2: CPXV012 peptide (CPX) prevents infection with MVA in different cell lines. Indicated cells were infected with MVA-eGFP (MOI 10) in the presence of 100 µg/ml peptide or DMSO. 18-20h after infection the amount of eGFP-positive cells was quantified using flow cytometry. Data were analyzed with one-way ANOVA followed by multiple comparisons Dunnett's test (the mean of each column was compared to that of the DMSO control). (* $p < 0.05$; ** $p < 0.01$; *** $p < 0.001$).

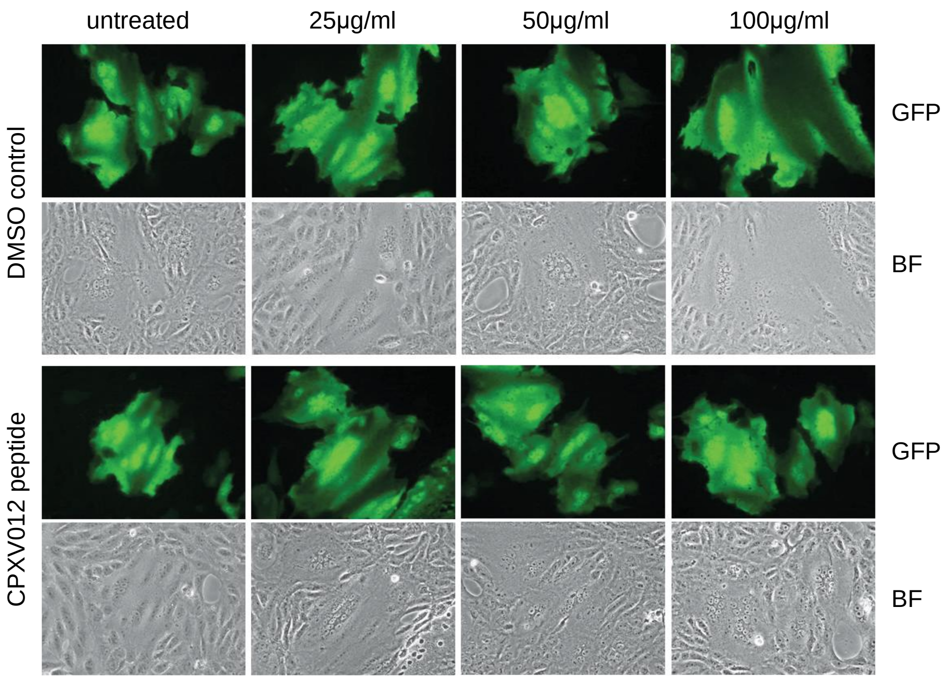

Figure S3: CPXV012 peptide does not affect infection with measles virus. Vero cells were left untreated or were treated with indicated concentrations of CPXV012 peptide or DMSO. Cells were infected with eGFP-expressing measles virus (MV-eGFP) (MOI 0.1). After maximum giant cell formation was observed (approximately at 48h post infection), fluorescence microscopy images were taken (magnification 10x). Results are representative of three independent experiments. BF: bright field.
